# Early ascorbic acid administration prevents vascular endothelial cell damage in septic mice

**DOI:** 10.3389/fphar.2022.929448

**Published:** 2022-10-06

**Authors:** Yutaro Madokoro, Chinatsu Kamikokuryo, Shuhei Niiyama, Takashi Ito, Satoshi Hara, Hiroshi Ichinose, Yasuyuki Kakihana

**Affiliations:** ^1^ Department of Emergency and Intensive Care Medicine, Kagoshima University Graduate School of Medical and Dental Sciences, Kagoshima, Japan; ^2^ Department of Biomedical Laboratory Sciences, Faculty of Life Sciences, Kumamoto University, Kumamoto, Japan; ^3^ School of Life Science and Technology, Tokyo Institute of Technology, Yokohama, Japan

**Keywords:** ascorbic acid, sepsis, tetrahydrobiopterin, endothelial dysfunction, syndecan

## Abstract

Oxidation of BH_4_, a cofactor of nitric oxide synthase (NOS), produces reactive oxygen species (ROS) through uncoupling of NOS and affects vascular endothelial dysfunction. Ascorbic acid (AsA) inhibits the oxidation of BH_4_ and reduces ROS. However, the kinetic changes of BH_4_ in sepsis and its effect on the kinetic changes in AsA administration therapy, as well as the appropriate timing of AsA administration for AsA therapy to be effective, are unclear. Mice with sepsis, induced by cecal ligation and puncture (CLP), were examined for the effect of AsA administration (200 mg/kg) on vascular endothelial cell dysfunction at two administration timings: early group (AsA administered immediately after CLP) and late group (AsA administered 12 h after CLP). Survival rates were compared between the early and late administration groups, and vascular endothelial cell damage, indicated by the dihydrobiopterin/tetrahydrobiopterin ratio, serum syndecan-1, and endothelial nitric oxide synthase, as well as liver damage, were examined. The early group showed significantly improved survival compared to the non-treatment group (*p* < 0.05), while the late group showed no improved survival compared to the non-treatment group. Compared to the non-treated group, the early AsA group showed less oxidation of BH_4_ in sepsis. Syndecan1, a marker of vascular endothelial cell damage, was less elevated and organ damage was reduced in the early AsA-treated group. In septic mice, early AsA administration immediately after CLP may protect vascular endothelial cells by inhibiting BH_4_ oxidation, thereby reducing organ dysfunction and improving survival.

## 1 Introduction

Sepsis is a life-threatening organ dysfunction caused by a dysregulated host response to infection (Beale et al., 2009). The World Health Organization reports that although sepsis mortality rates have declined in recent decades, it still causes 11 million deaths annually (Rudd et al., 2020). Currently, there is no definitive treatment for sepsis, and the recommended treatment includes early detection, early antibiotic administration, appropriate infusion therapy, and optimal timing of vasopressor administration; however, the mortality rate remains high (Evans et al., 2021).

Since vascular endothelial dysfunction is associated with the pathological progression of sepsis, maintaining vascular endothelial function is attracting attention as a new therapeutic strategy for sepsis (Becker et al., 2010; Ince et al., 2016; Uchimido et al., 2019; Lupu et al., 2020). The vascular endothelium is covered with glycocalyx, a gel-like protective layer, which plays an important role in vascular endothelial function, including vascular permeability, anticoagulation, and nitric oxide (NO) production from endothelial nitric oxide synthase (eNOS). The glycocalyx is composed of syndecan-1, heparan sulfate, hyaluronic acid, etc. In sepsis, reactive oxygen species (ROS), tumor necrosis factor-alpha (TNF-alpha), interleukin-1beta (IL-1beta), and other factors cause the glycocalyx to be shed, resulting in increased vascular permeability and coagulation and decreased NO production (Uchimido et al., 2019).

Tetrahydrobiopterin (BH_4_) is produced from guanosine triphosphate and acts as an essential cofactor for various enzymes. BH_4_ is easily oxidized to dihydrobiopterin (BH_2_), and the binding affinities of BH_4_ and BH_2_ to eNOS are equal. NO is produced when BH_4_ binds to eNOS, whereas superoxide is produced when BH_2_ binds to eNOS, namely uncoupling of eNOS (Vásquez-Vivar et al., 2002). The relationship between BH_4_ and vascular endothelial function has been reported in various diseases such as hypertension (HT), diabetes mellitus (DM), and atherosclerosis. In addition, there are indications that oxidation of BH_4_ affects endothelial dysfunction in all these diseases (Kolluru et al., 2012; Ismaeel et al., 2020; Kim and Han, 2020). Although it has been considered that the lack of BH_4_ and/or the increase of BH_2_ are the causes of the uncoupling of NOS, it has recently been reported that the BH_2_/BH_4_ ratio is more related to ROS generation and vascular endothelial dysfunction than the absolute value of BH_4_ or BH_2_ (Crabtree et al., 2008; Takeda et al., 2009; Pathak et al., 2014; Ismaeel et al., 2020). Inhibiting the oxidation of BH_4_ and preventing the increase in BH_2_/BH_4_ ratio are important for maintaining vascular endothelial function.

Ascorbic acid (AsA), also known as vitamin C, is an important antioxidant that prevents the oxidation of various substances, including BH_4_ (Heller et al., 2001). AsA has important effects on the maintenance of vascular endothelial functions, with multiple pathways known to exert vascular endothelial protection, including inhibition of BH_4_ oxidation (May and Harrison, 2013). In addition, there have been recent studies suggesting the efficacy of AsA administration in sepsis. Vitamin C levels are decreased in critically ill patients, such as those with sepsis (Carr et al., 2017). In sepsis, AsA administration has been reported to improve survival and protect microvascular functions (Tyml et al., 2008; Fowler et al., 2014; Zabet et al., 2016; Lv et al., 2021). It has been reported that AsA has multiple mechanisms of action for sepsis, one of which is by inhibiting BH_4_ oxidation (Moskowitz et al., 2018).

However, while AsA inhibits the oxidation of BH_4_, it does not reduce BH_2_ to BH_4_ (Vásquez-Vivar et al., 2001). After BH_4_ has been oxidized to BH_2_, the effect of AsA administration on this mechanism cannot be expected and may be limited by the timing of AsA administration. We hypothesized that administration of AsA at the optimal timing, before the BH_2_/BH_4_ ratio increases, would protect the glycocalyx and improve sepsis survival. In our preliminary experiments using the cecal ligation and puncture (CLP) model mice, the BH_2_/BH_4_ ratio showed an upward trend from 6 h after the onset of sepsis. Therefore, we examined the unclear kinetics of when the BH_2_/BH_4_ ratio changes in sepsis and how AsA administration before oxidation to BH_2_ affects the BH_2_/BH_4_ ratio and influences survival.

## 2 Methods

### 2.1 Animals

Adult C57BL/6 mice (9–11-week-old males) weighing 25 g were obtained from Kyudo (Fukuoka, Japan), housed under standard environmental conditions, and maintained at 23 ± 1°C with a 12-h light/dark cycle. All animal experiments were conducted under the rules approved by the Institutional Animal Care and Use Committee of Kagoshima University (approval number MD18126). As this was an animal study, consents for participation and publication were not applicable. We carried out the study in compliance with the ARRIVE guidelines (https://arriveguidelines.org) and the Guidelines for the Proper Conduct of Animal Experiments established by the Science Council of Japan.

### 2.2 CLP

As previously reported, septic shock was induced with reference to the high grade model CLP in the article by Rittirsch et al. with slight modifications (Rittirsch et al., 2009). Briefly, mice were anesthetized with isoflurane, and the mouse cecum was ligated with a 3-0 silk suture and punctured in one place with a 21-gauge needle. The cecum was retracted into the abdominal cavity, and the incision was sutured with 3-0 nylon. Only open and closed abdominal procedures were performed for sham-operated mice without CLP. During each experiment, blood, liver, and heart tissues were collected and analyzed under inhalation anesthesia. At 6, 12, and 24 h after CLP and sham operation, the blood of mice was collected by inferior vena cava puncture, after which the animals were sacrificed. At the same time point, the myocardium and liver were collected. Blood samples were centrifuged at 2,000 g for 10 min to collect plasma and stored at −80°C until analysis. After the operation, buprenorphine (0.05 mg/kg) was repeatedly administered every 12 h by subcutaneous injection.

### 2.3 Experimental design

#### 2.3.1 Survival experiment: CLP vs. CLP + AsA (early)

**FIGURE 1:**
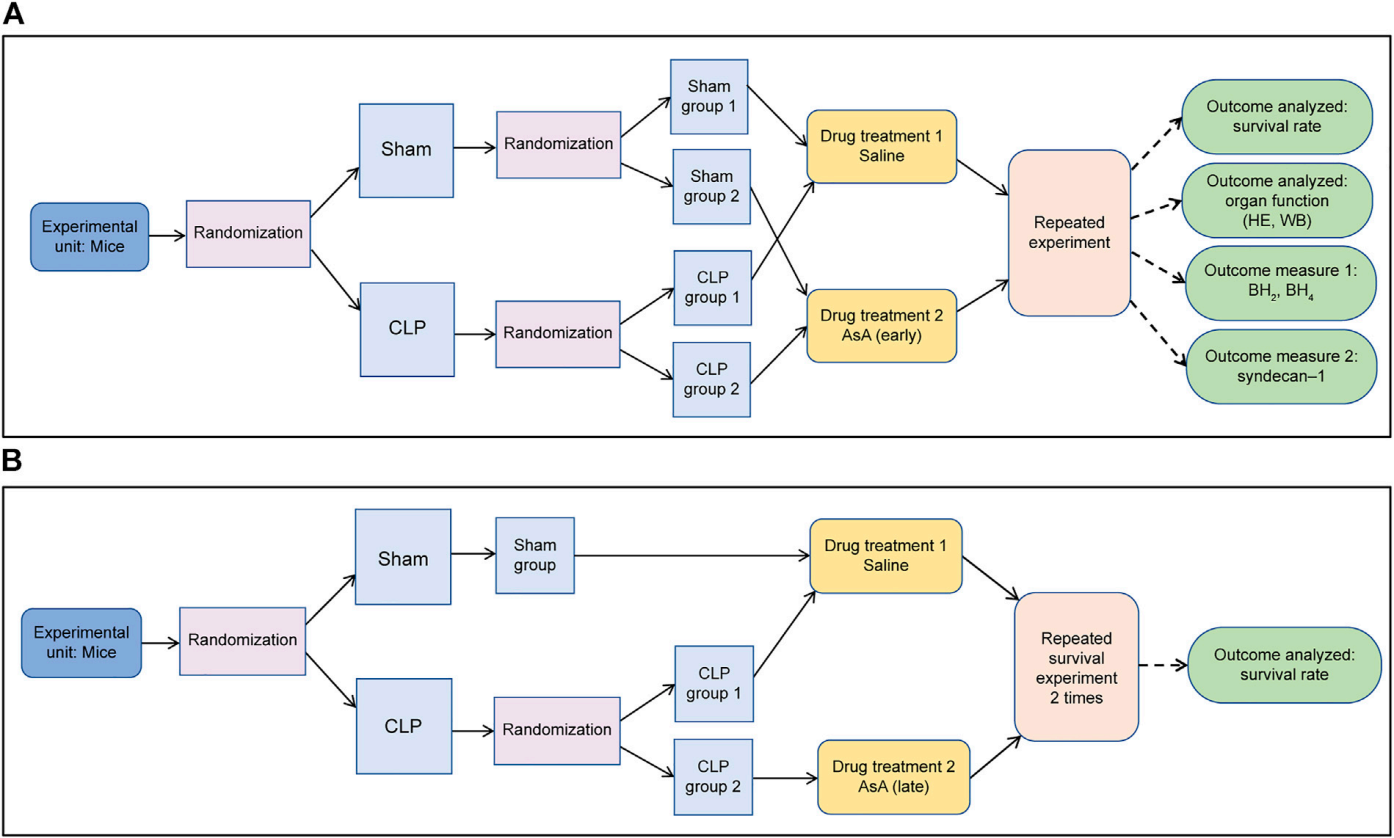
**(A)** The surgeon was unaware of the drug treatment that the animals received from the start of the experiment until after the injection. **(B)** The surgeon was unaware of the drug treatment that the animals received from the start of the experiment until after the injection.

Mice were randomized into the following groups: 1) sham (*n* = 10); 2) sham + AsA (early) (*n* = 10); 3) CLP (*n* = 10), and 4) CLP + AsA (early) groups (*n* = 9). The group that had not received AsA was given the same amount of NS needed to dissolve the AsA in the AsA group. These mice received 40 ml/kg of normal saline or AsA (200 mg/kg) by subcutaneous injection immediately after the operation and were monitored for 72 h (Figure 1A; Table 1). In the study, n refers to the number of animals. The numbers for each group were taken from similar experiments reported in the literature. AsA was administered at the optimal dose of 200 mg/kg/day for this experiment, as its effectiveness has been demonstrated previously in septic mice (Wu et al., 2003; McKinnon et al., 2007; Kim et al., 2015; Jensen et al., 2021).

**TABLE 1:** Number of mice per group. ∗These groups originally numbered 10 animals, but the feces were hard and the severity of the disease could not be assessed; thus, we excluded 2 mice from the study.

	Injection time after operation	Operation	Treatment	N =
Early group	immediately	Sham	NS	10
			AsA	10
		CLP	NS	10
			AsA	9*
Late group	After 12hr	Sham	NS	10
		CLP	NS	10
			AsA	9*

#### 2.3.2 Survival experiment: CLP vs. CLP + AsA (late)

Mice were randomized into the following groups: 1) sham (*n* = 10), 2) CLP (*n* = 10), and 3) CLP + AsA (late) (*n* = 9). The group that had not received AsA was given the same amount of NS needed to dissolve the AsA in the AsA group. Mice received 40 ml/kg of normal saline by subcutaneous injection immediately after the operation, and 10 ml/kg of normal saline or AsA (200 mg/kg) by subcutaneous injection at 12 h after the operation. The mice were monitored for 72 h (Figure 1B; Table 1).

#### 2.3.3 Measurement of BH_4_ and BH_2_ and calculation of BH_2_/BH_4_

BH_4_ is a substance that oxidizes easily, and oxidation was prevented by adding 0.2% dithioerythritol (a final concentration). BH_4_ and BH_2_ were measured separately by the post-column oxidation method using high-performance liquid chromatography with a fluorescence detector (Tani and Ohno, 1993). The plasma samples (100 μl) were deproteinized by adding 25 µl of 1 M perchloric acid containing 0.5 mM EDTA, followed by centrifugation. The supernatants were filtered through a 0.2-μm filter. The BH_2_/BH_4_ ratio was calculated by dividing BH_2_ by BH_4_. After 6, 12, and 24 h of operation in the sham + NS group, the number of mice in each group was 7, 4, and 4, respectively. In the CLP+ NS group were 8, 6, and 7, respectively. Finally, the CLP+ AsA group was 7, 7, and 7, respectively. The number of mice in the control group was 5 (Figure 1A; Table 2).

**TABLE 2:** Outcome measurement. ∗The numbers of each group deviated because some individuals died during the course of the study. Including the dead mice, the total is 152.

	Operation	Collecting time after operation	Treatment	N =
BH2,BH4	Control		None	5
	Sham	6 h	NS	7
		12 h		4
		24 h		4
	CLP	6 h	NS	8
		12 h		6
		24 h		7
	CLP + AsA	6 h	AsA	7
		12 h		7
		24 h		7
Syndecan-1	Control		None	4
	Sham	6 h	NS	7
		12 h		6
		24 h		7
	CLP	6 h	NS	7
		12 h		12
		24 h		11
	CLP + AsA	6 h	AsA	7
		12 h		7
		24 h		7

#### 2.3.4 Measurement of Syndecan-1

Plasma syndecan-1 levels were measured using a Murine CD138 ELISA Kit (Diaclone, France). The number of mice in each group after 6, 12, and 24 h of operation in the sham + NS group was 7, 6, and 7, respectively. In the CLP+ NS group, the number was 7, 12, and 11, respectively. Finally, the CLP+ AsA group was 7, 7, and 7, respectively (Figure 1A and Table 2).

#### 2.3.5 Western blotting analysis

The heart was homogenized in a buffer solution (T-PER Tissue Protein Extraction Reagent; Thermo Scientific, Rockford, USA). The extracted proteins were quantified (TaKaRa BCA Protein Assay Kit, Takara Holdings Inc, Japan), and the amount of protein to be applied to the gel was adjusted. The protein samples (1 μg of protein) were electrophoresed on 10% SDS-PAGE and transferred to the PVDF membrane. The membrane was blocked for 1 h (BLOCK ACE^®^, MEGMILK SNOW BRAND, Japan) and incubated with primary antibodies (eNOS, 1:1,000, Purified Mouse Anti-eNOS/NOS Type III, BDbioscience, USA; GAPDH, 1:20,000, Anti-GAPDH Loading Control ab8245, Abcam, UK) at 4°C overnight. After washing with Phosphate Buffered Saline with Tween (PBST) buffer, the membranes were incubated with horseradish peroxidase (HRP)-conjugated secondary antibody (1:5,000, Goat Anti-Mouse IgG H&L HRP ab205719, Abcam, UK) for 1 h at room temperature. Blots were washed with PBST, and immunoreactive bands were detected using an enhanced chemiluminescence system (ImmunoStar^®^, FUJIFILM Wako Chemical Corporation, Japan) (Figure 1A). Optical density for individual bands was examined using the Fluor Chem FC2 (Cell Biosciences, Santa Clara, CA, United States of America). The densitometry ratios of eNOS to GAPDH were then computed.

### 2.3.6 Histologic examination

Liver tissue specimens were fixed in 10% formalin and embedded in paraffin. They were stained with hematoxylin and eosin to evaluate the degree of injury (Figure 1A).

### 2.4 Statistical analysis

Survival rates were analyzed using the Kaplan–Meier method. Survival times were compared using the log-rank test. Data are expressed as mean ± standard error. The Kruskal–Wallis test was used to detect differences between the groups. The Bonferroni method was used for the post hoc test of this statistic. Student’s t-test was used for comparisons between the two groups of quantified western blots. Significant differences were considered if the *p*-value was <0.05.

## 3 Results

### 3.1 Early administration of AsA improved the survival rate of septic mice

**FIGURE 2:**
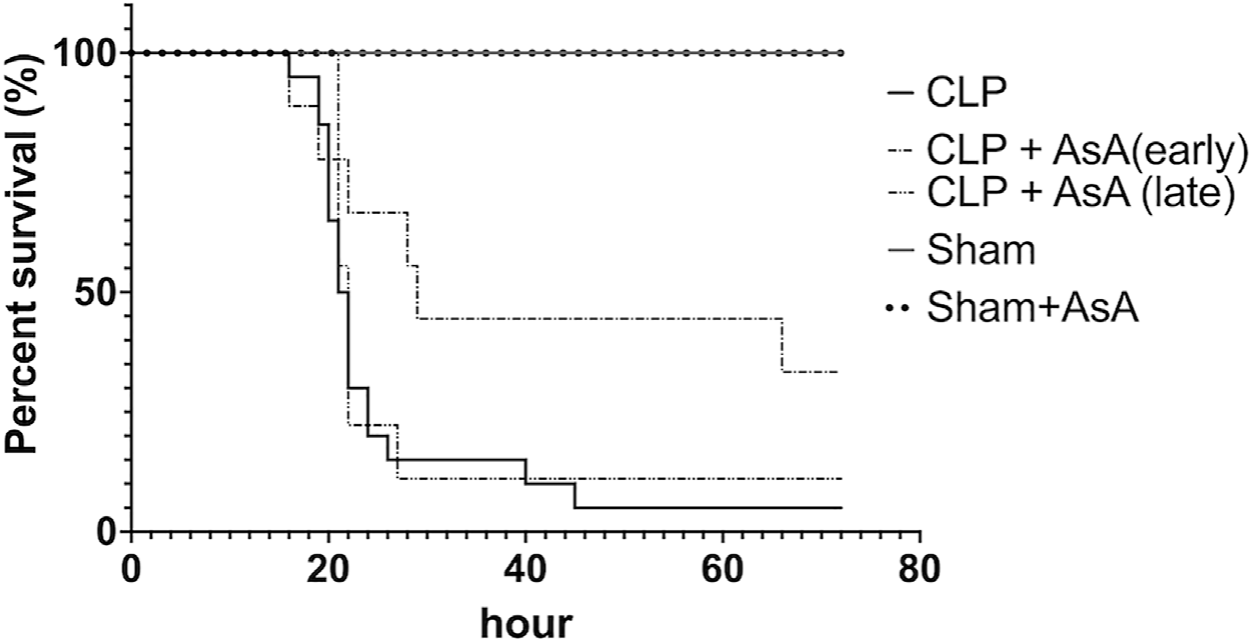
Mice subjected to CLP, as described in the Methods section, for 72 h survival study. In the CLP + AsA (early) group, AsA was injected subcutaneously immediately after the operation (*n* = 9). In the CLP + AsA (late) group, AsA was injected subcutaneously 12 h after the operation (*n* = 9). The group that had not received AsA was given the same amount of NS needed to dissolve the AsA in the AsA group. Although the timing of saline administration in the CLP group differed between the late and early groups, they were compared with the other groups as the same group because the severity of the disease did not change. The CLP + AsA (early) mice group had a significantly prolonged survival rate compared to the CLP group. *p < 0.05 versus CLP. CLP + AsA (late) mice showed no difference in the survival rate from the CLP group. *p < 0.05 versus CLP. Abbreviations: AsA, ascorbic acid; CLP, cecal ligation and puncture.

We compared the survival rates after operation between sham, sham + AsA (early), CLP, and CLP + AsA (early) (Figure 2). None of the CLP mice used in this study survived to 45 h after the operation. All sham + NS and sham + AsA (early) mice survived for 72 h. In the CLP + AsA (early) mice group, 3 of 9 mice survived after the operation (33%).

The CLP + AsA (early) mice group showed significantly higher survival rates than the CLP mice group.

Second, we compared the survival rates after operation among sham, CLP, and CLP + AsA (late) groups (Figure 2). AsA (Late) mice group received 40 ml/kg of normal saline by subcutaneous injection immediately after the operation, and AsA (200 mg/kg) by subcutaneous injection at 12 h after the operation. All sham mice survived for 72 h. In the CLP and CLP + AsA (late) mice groups, 1 of 9 mice survived after the operation (11%). The CLP + AsA (late) mice group showed no difference in survival rates compared to the CLP mice group.

### 3.2 BH2/BH4 ratio increased 6 h after the operation and continued to increase over time. Early administration of AsA prevented an increase in BH2/BH4 ratio

To elucidate the dynamics of BH_4_ and BH_2_ in CLP-induced sepsis and how the dynamics of the BH_2_/BH_4_ ratio change with the administration of AsA (early), we measured BH_4_ and BH_2_ and then calculated BH_2_/BH_4_. Serum BH_4_ and BH_2_ levels were determined in CLP and sham mice at 6, 12, and 24 h after the operation.

**FIGURE 3:**
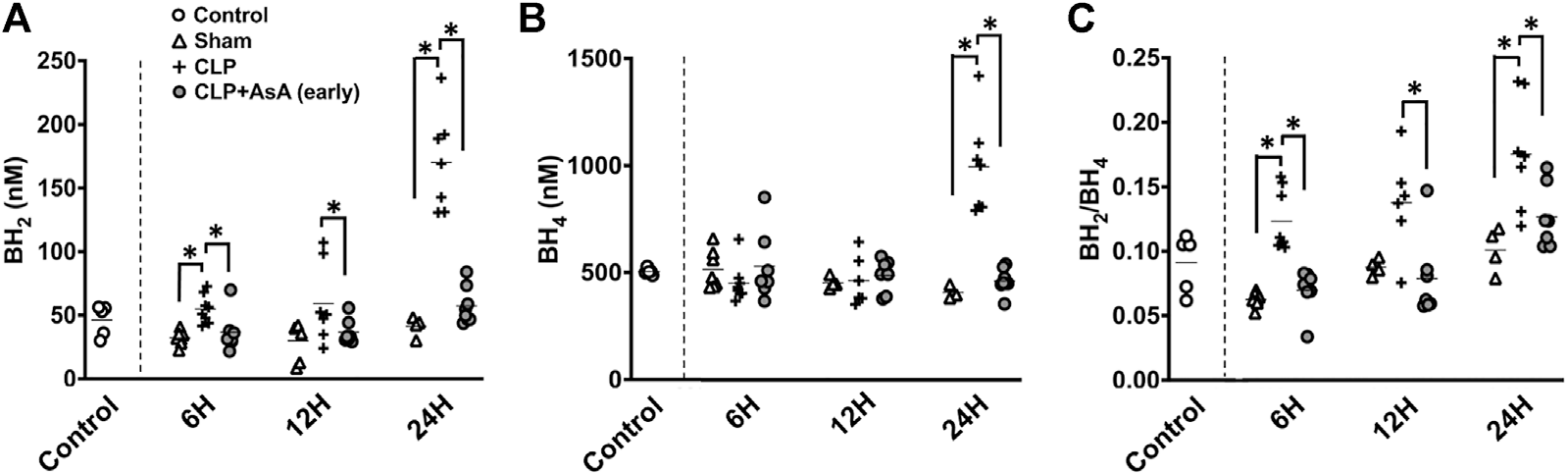
**(A)** BH_2_ concentration in the plasma was measured by HPLC. At 6 and 24 h after the operation, CLP group had significantly elevated BH_2_ concentration compared to CLP+ AsA (early) and Sham groups. At 12 h after the operation, CLP group had significantly elevated BH_2_ concentration compared to CLP+ AsA (early) group. Error bars represent SE. *p < 0.05. **(B)** BH_4_ concentration in the plasma was measured by HPLC. At 24 h after the operation, CLP group had significantly elevated BH_4_ concentration compared to CLP+ AsA (early) and Sham groups. At 6 and 12 h after the operation, there was no significant differences between the groups. Error bars represent SE. *p < 0.05. **(C)** BH_2_/BH_4_ ratio was calculated by dividing BH_2_ by BH_4_. At 6 and 24 h after the operation, CLP group had significantly elevated BH_2_/BH_4_ ratio compared to CLP+ AsA (early) and Sham groups. At 12 h after the operation, CLP group had significantly elevated BH_2_/BH_4_ ratio compared to CLP+ AsA (early) group. Error bars represent SE. *p < 0.05. Abbreviations: AsA, ascorbic acid; CLP, cecal ligation and puncture; BH4, tetrahydrobiopterin; BH2, dihydrobiopterin; SE, standard error; HPLC, high-performance liquid chromatography.

Both BH_4_ and BH_2_ showed a significant increase 24 h after the operation (Figure 3). The ratio of BH_2_ to BH_4_ was significantly elevated in the CLP group compared to the early AsA group starting at 12 h.

Syndecan-1 levels increased after 12 h, but early administration of AsA suppressed this increase. The expression of eNOS in myocardial tissues was also maintained by the early administration of AsA.

Syndecan-1 level and eNOS expression in myocardial tissues were measured to evaluate whether early administration of AsA protects vascular endothelial cells.

**FIGURE 4:**
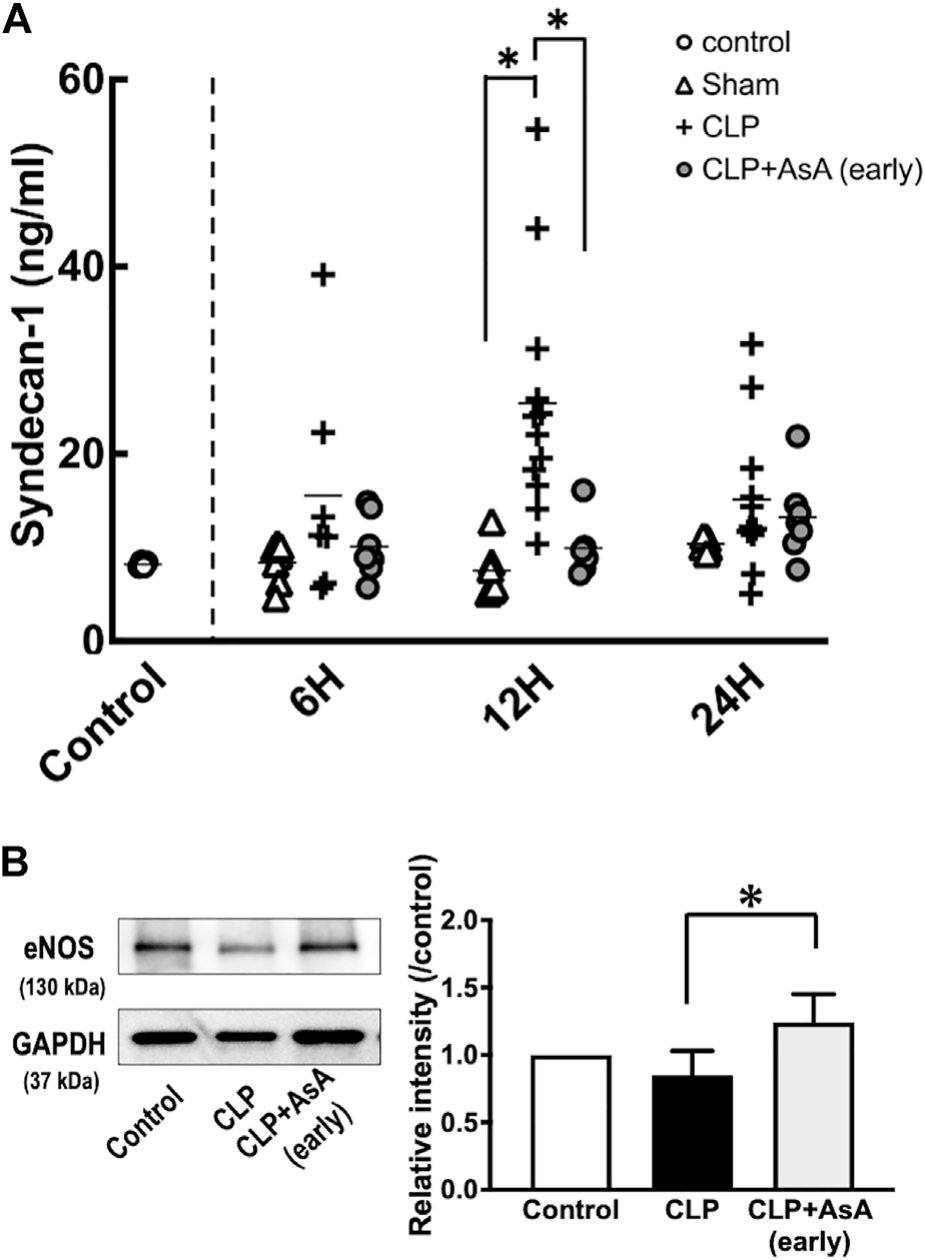
**(A)** Plasma syndecan-1 was measured by ELISA kit. At 12 h after the operation, CLP group had significantly elevated syndecan-1 level compared to the CLP+ AsA (early) and Sham groups. Error bars represent SE. *p < 0.05. **(B)** All samples were taken 12 h postoperatively. The expression of eNOS (130 kDa) in the heart was measured by western blotting; it was decreased in the CLP group but maintained in the early AsA group. eNOS was quantified and compared between the CLP and CLP + AsA (early) groups (*n* = 4, 4). For the quantitative experiments, measurements were repeated multiple times. eNOS expression was significantly maintained in the AsA (early) group. *p < 0.05. Abbreviations: AsA, ascorbic acid; CLP, cecal ligation and puncture; eNOS, endothelial NO synthase; SE, standard error.

Syndecan-1 at 12 h was significantly higher in the CLP group than in the early AsA group (Figure 4). The expression of eNOS was measured to evaluate vascular endothelial cells and was assessed in myocardial tissue 12 h after operation. Four samples from the normal, CLP, and CLP + AsA (early) groups were collected and evaluated by western blotting. eNOS expression was lower in the CLP group than in the normal group. Compared to the CLP group, the early AsA group maintained eNOS expression.

### 3.3 Liver organ damage was reduced by early AsA administration

Finally, liver tissue was stained with hematoxylin and eosin and observed under a microscope to evaluate organ damage due to sepsis. Each sample was collected 12 h after the operation.

**FIGURE 5:**
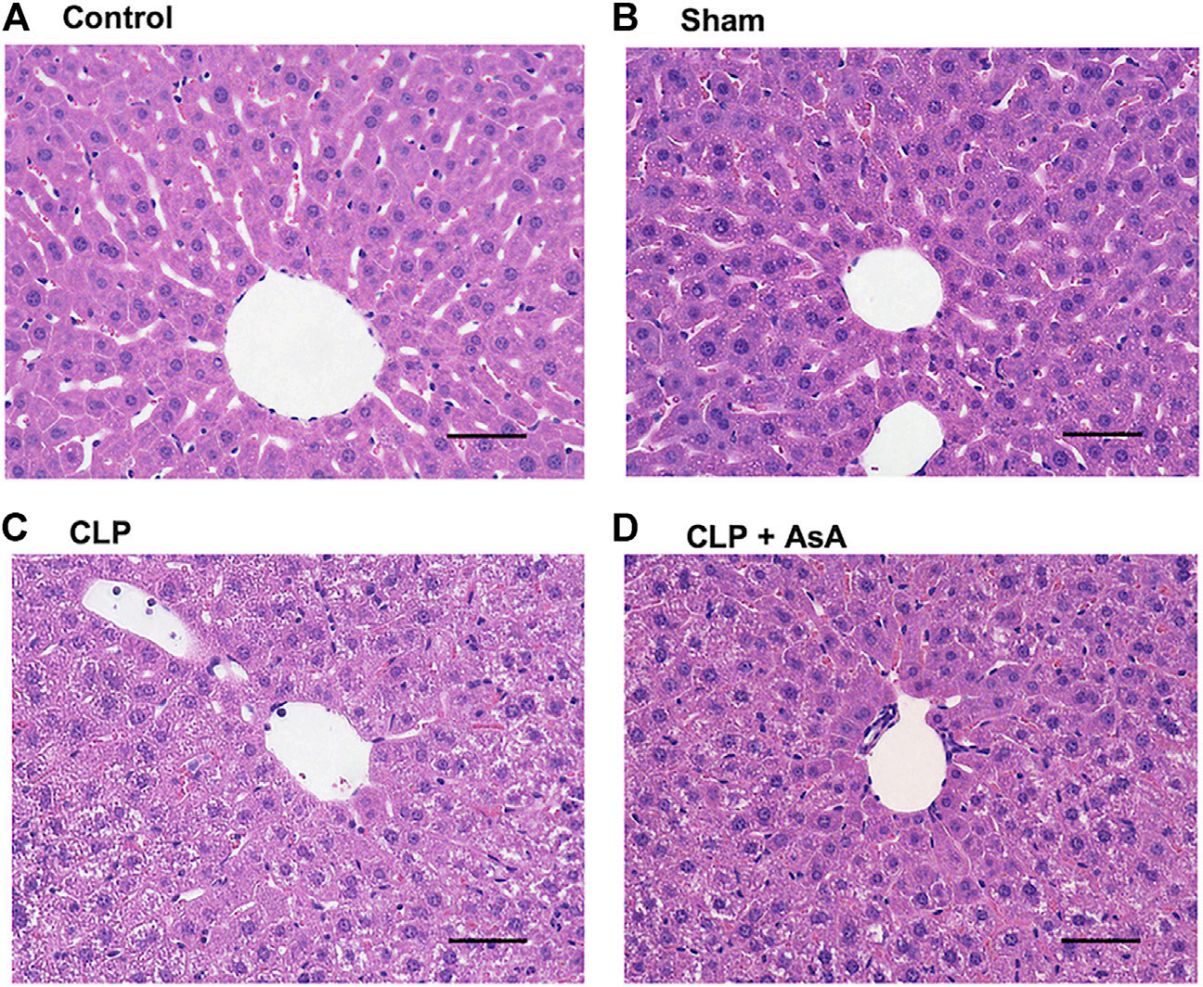
Histopathological examination of the liver of CLP-treated and untreated mice. **(A,B)** Normal histology of liver tissues obtained from sham and control mice. **(C)** Representative CLP-induced liver damage. **(D)** Representative liver of CLP mice treated with early AsA administration. Sham and CLP mice were killed 12 h after the operation. Original magnification, ×40. Abbreviations: AsA, ascorbic acid; CLP, cecal ligation and puncture

No histological differences were observed between the control and sham mice. In CLP mice, the arrangement of hepatocytes was markedly disorganized (Figure 5). In contrast, hepatocyte disarrangement was reduced in CLP + AsA (early) mice, although not as orderly as in the control and sham mice.

## 4 Discussion

This study showed that early administration of AsA might contribute to improved survival of septic mice (Figure 2). In particular, we found that the optimal timing of AsA administration should be early in the disease, before the BH_2_/BH_4_ ratio increases. Although the efficacy of AsA in sepsis is still under discussion, our study suggests that it may be more effective if administered at the optimal time. Here, we discuss the effect of early AsA administration on BH_4_ dynamics and the optimal timing of AsA administration.

BH_4_ plays an important role in maintaining vascular endothelial function by producing NO as a cofactor for eNOS. It also plays a role in exacerbating the pathogenesis of sepsis by overproducing NO through inducible nitric oxide synthase (iNOS) expression and producing free radicals through eNOS uncoupling (Ince et al., 2016; Dolmatova et al., 2021). The elevation of the BH_2_/BH_4_ ratio is associated with ROS development. It has been reported to correlate with vascular endothelial dysfunction in various diseases, such as HT, DM, and peripheral arterial disease. (Crabtree et al., 2008; Crabtree and Channon, 2011; Ismaeel et al., 2020). However, there are few reports on the dynamics of BH_4_ in the acute phase and the relationship between the BH_2_/BH_4_ ratio and vascular endothelial function in sepsis. In our septic mice experiment, the BH_2_/BH_4_ ratio was shown to increase as early as 6 h after CLP, indicating that the BH_2_/BH_4_ ratio increases in sepsis, vascular endothelial dysfunction is associated with exacerbation, as in other diseases (Figure 3C).

In case of an increase in BH_2_/BH_4_ ratio, eNOS undergoes an uncoupling reaction, which produces superoxide, instead of NO, that reacts to form peroxynitrite (ONOO-), a powerful oxidant (Stuehr et al., 2001; Vásquez-Vivar et al., 2002; Alkaitis and Crabtree, 2012). Peroxynitrite is one of the most powerful ROS, making it a major cause of vascular endothelial dysfunction. It is considered that correcting the BH_2_/BH_4_ ratio can inhibit peroxinitrite production and protect the vascular endothelium (Bendall et al., 2014). Therefore, attempts to correct the relative lack of BH_4_ by supplementing BH_4_ to prevent uncoupling of eNOS and maintain endothelial function have been reported in several diseases such as patients of coronary risk factors, HT, DM, and ischemia reperfusion (Heitzer et al., 2000; Mayahi et al., 2007; Porkert et al., 2008). In sepsis, BH_4_ administration reportedly improved microcirculation, circulatory indices, and survival rate in a sheep sepsis model (He et al., 2012; Dumbarton et al., 2017). However, conflicting studies have shown that inhibition of BH_4_ production improves sepsis mortality (Chuaiphichai et al., 2016). Thus, there are conflicting reports on the administration of BH_4_ for sepsis. One reason why BH_4_ administration is not effective in the acute phase of sepsis is that BH_4_ is overproduced in the acute phase of sepsis, and the overproduction of BH_4_ may be related to its autoxidation to BH_2_ (Kirsch et al., 2003). Another possible cause is that exogenous BH_4_ is converted to BH_2_ after administration (Cunnington et al., 2012). Since treatment that inhibits BH_4_ oxidation may be more effective than administration of BH_4_ to improve the BH_2_/BH_4_ ratio in the acute phase of sepsis, when BH_4_ production is overproduced, we focused on the antioxidant AsA.

AsA has long been used as an antioxidant in BH_4_ measurement methods (Tani and Ohno, 1993). In sepsis, the administration of AsA alone and the simultaneous administration of vitamin B1 and hydrocortisone have been widely studied. The simultaneous administration of vitamin B1 and hydrocortisone, in particular, has attracted attention as a type of metabolic therapy such as Hydrocortisone, ascorbic acid, and thiamine therapy (Marik et al., 2017; Marik, 2018; Fowler et al., 2019; Kim et al., 2020). In clinical practice, administration of AsA, a reducing agent, has been reported to inhibit the oxidation of BH_4_ (Mortensen and Lykkesfeldt, 2014). In our study, the BH_2_/BH_4_ ratio increased at 6 h after operation, and AsA administration immediately after operation significantly suppressed the increase in BH_2_/BH_4_ ratio (Figure 3C). In the AsA non-administered group, an increase in serum syndecan-1 level, an indicator of endothelial cell damage, and decreased eNOS expression, an indicator of endothelial cell protection, were observed at 12 h after operation (Figures 4A,B).

On the other hand, in the early AsA group immediately after the operation, both serum syndecan-1 level and eNOS expression level showed protective effects on vascular endothelium (Figures 4A,B). In addition, in the non-AsA-treated group, where syndecan-1 was elevated and eNOS expression was decreased, organ damage occurred after 12 h. In contrast, early AsA administration suppressed syndecan-1 elevation and reduced organ damage in the group where eNOS expression was maintained (Figure 5). Serum syndecan-1 is a known indicator of vascular endothelial damage that correlates with coagulation disorders associated with sepsis prognoses, such as persistent thrombocytopenia and disseminated intravascular coagulation (DIC) (Ostrowski et al., 2015; Hatanaka et al., 2021). The suppression of syndecan-1 elevation in the early AsA group suggests that vascular endothelial cell damage suppression resulted in less sepsis-induced organ damage. Since AsA inhibits the oxidation of BH_4_, the increase in BH_2_/BH_4_ ratio was suppressed when AsA was administered immediately after operation. The fact that the survival rate did not improve when AsA was administered after 12 h (late group) in the survival experiment may be due to the late timing of administration since AsA does not have the effect of reducing BH_2_ to BH_4_ (Figure 2) (Vásquez-Vivar et al., 2001).

Several recent studies have shown no positive effect of AsA administration in septic shock patients, so it remains controversial whether AsA should be administered to these patients (Fujii et al., 2020; Moskowitz et al., 2020; Scholz et al., 2021). Some studies have cited delayed administration as a limiting factor to obtaining a good effect of AsA in septic shock (Moskowitz et al., 2020). Since the reduction of BH_2_ to BH_4_ is not expected to be effective, making a difference in the survival results depending on the timing of AsA administration, as shown in our present experiment with septic mice, the studies reporting no effect of AsA therapy may be related to the fact that AsA was administered after the BH_2_/BH_4_ ratio was already elevated. The optimal timing of AsA administration for sepsis will become more important, as an experiment is currently planned to test the efficacy of early AsA administration in the emergency room for septic patients (Vandervelden et al., 2021).

This study has several limitations. First, it is unclear whether the results from this septic mouse experiment would be similar to those of human sepsis. Changes in BH_4_ and BH_2_ over time may differ between humans and mice. Second, because mice can synthesize AsA in their bodies, their bodies’ dynamics of AsA concentration may be different from those of humans. The optimal dosage needs to be discussed in both human and animal studies. Third, we have not measured intracellular BH4; some experiments have measured BH_4_ and BH_2_ in cells rather than in plasma as in our study. Although we consider that the kinetics of the two move generally in parallel, it may have been necessary to measure the BH_2_/BH_4_ ratio in vascular endothelial cells in order to correlate ROS production (eNOS function) in vascular endothelial cells with the BH_2_/BH_4_ ratio in plasma.

Moreover, in this study, we only mentioned the protective effect of AsA on vascular endothelial cells by suppressing the increase in BH_2_/BH_4_ ratio. Still, AsA has additional effects, such as catecholamine production, adrenocorticotropic hormone production, and direct scavenging of free radicals, which may improve the prognosis of sepsis through various pathways (Patak et al., 2004; Padayatty et al., 2007; Moskowitz et al., 2018; Obi et al., 2020). However, our present findings indicate that the timing of AsA administration affects prognosis and that the BH_2_/BH_4_ ratio is related to the mechanism of septic shock.

## 5 Conclusion

In the septic mice, an increase in the BH_2_/BH_4_ ratio, which causes vascular endothelial cell damage, occurred 6 h after the disease onset. In the present study, we suggest that administration of AsA at an earlier time before the increase in the BH_2_/BH_4_ ratio, suppressed the increase in the BH_2_/BH_4_ ratio and contributed to the improved prognosis of the septic mice. In the future, the time course of the BH_2_/BH_4_ ratio in septic patients should be evaluated to determine the optimal timing of AsA administration.

## Data Availability

The original contributions presented in the study are included in the article/supplementary material, further inquiries can be directed to the corresponding author.
